# Seven novel genetic variants in a North Indian cohort with classical homocystinuria

**DOI:** 10.1038/s41598-020-73475-5

**Published:** 2020-10-14

**Authors:** Rajdeep Kaur, Savita V. Attri, Arushi G. Saini, Naveen Sankhyan, Satwinder Singh, Mohammed Faruq, V. L. Ramprasad, Sheetal Sharda, Sakthivel Murugan

**Affiliations:** 1 Department of Pediatrics, Postgraduate Institute of Medical Education and Research (PGIMER), Chandigarh, 160012 India; 2 Institute of Genomics and Integrative Biology (IGIB), New Delhi, India; 3 MedGenome Labs Ltd., Bangalore, India

**Keywords:** Biochemistry, Genetics, Diseases, Neurology

## Abstract

Classical homocystinuria is the most common cause of isolated homocystinuria. The variants of the *CBS* gene remain unidentified in Indian children with this disorder. Based on the hallmark clinical features, family history, and/or biochemical clues for classical homocystinuria, 16 children below the age of 18 years were evaluated by Sanger sequencing of the coding exons of *CBS* gene with flanking intronic regions. The common C677T variant of the *MTHFR* gene was also screened by restriction fragment length polymorphism. Fifteen children were clinically suspected of having classical homocystinuria and one asymptomatic child with positive family history. Only seven children had biochemical features of classical homocystinuria. Sanger sequencing of the *CBS* gene confirmed 15 different pathogenic or likely pathogenic variants in 14 cases. Of these, seven variants were novel (three frameshift deletions, two nonsense, one missense, one splice site variant) and were predicted to be deleterious by Mutation Taster software. Seven cases were homozygous, another six were compound heterozygous, and one case was single heterozygous in the study. None of the three most frequent mutations reported worldwide viz., I278T, G307S, and IVS 11-2A>C were found in our cohort. No variants were detected in the exons 2, 8, 12, and 14 as compared to reported literature. Eleven out of 15 variants were associated with the conserved catalytic domain of the CBS polypeptide. The *MTHFR* polymorphism C677T was observed in heterozygous state in six cases. Our study reports the detailed genotype and seven novel variants in the *CBS* gene, causing classical homocystinuria in Indian children. The genetic analysis will help to offer accurate genetic counseling, prenatal diagnosis, and development of mutation-based novel therapeutic strategies.

## Introduction

Homocystinuria refers to the excretion of homocystine in the urine. It results from either a nutritional deficiency or an inherited defect in the enzymes involved in the remethylation and transsulfuration pathways of methionine metabolism. Classical homocystinuria (MIM#236200) is an autosomal recessive disorder due to the deficiency of cystathionine-β-synthase (CBS) enzyme (EC#4.2.1.22). It is the most common cause of homocystinuria. CBS is a pyridoxal-5′-phosphate-dependent enzyme which catalyzes the conversion of homocysteine and serine to cystathionine in the transsulfuration pathway of homocysteine metabolism to form cysteine^1^. The characteristic amino acid profile of CBS deficiency shows high levels of homocysteine and methionine, and low levels of cystathionine and cysteine in the blood and urine^2^. The disorder presents with a myriad of clinical symptoms, mainly related to the four organ systems, i.e., ocular, nervous, skeletal, and vascular system. The global incidence of classical homocystinuria is estimated to be 1 in 344,000^3^. A higher incidence has been reported from Ireland (1 in 65,000)^4^ and Qatar (1 in 1800)^5^ due to higher consanguinity. The actual incidence in India is unknown.

The CBS enzyme in humans, encoded by the *CBS* gene (genomic ref seq NG_008938.1, coding DNA Ref seq. NM_000071.2), has been mapped to chromosomal location 21q22.3^6^ with 17 exons and 2605 base pairs. The resultant protein is homotetrameric formed from 63 kD subunits, each consisting of 551 residues^7^. A total of 195 different *CBS* gene variants have been reported to date (as per the Human Gene Mutation Database). The I278T (c.833T>C) variation is most frequently reported worldwide^8^, accounting for 55% of the mutant alleles in the Netherlands^9^. The heterogeneous distribution of *CBS* gene variants was observed from different geographical locations^10–14^. The spectrum of *CBS* gene variants in Indian children with classical homocystinuria has not been reported to date.

## Patients and methods

### Study participants

Sixteen children below 18 years of age with a clinical and biochemical diagnosis of classical homocystinuria, and being followed up in the Pediatric Neurology clinic of the Department of Pediatrics at the Postgraduate Institute of Medical Education and Research, Chandigarh, India, were enrolled. Clinical features considered were the presence of severe myopia, ectopia lentis, intellectual disability, marfanoid features, or thromboembolism events. Short and tall stature was defined as height < 3rd percentile and > 97th percentile^15^ of the normal population growth chart; respectively, as per Indian Academy of Pediatrics guidelines^16^. The biochemical markers considered were an elevated methionine, with high plasma or urine homocysteine levels on two or more occasions. All newly diagnosed children with homocystinuria were first supplemented with oral B_12_ and folate to rule out a nutritional vitamin deficiency. All the experimental protocols were approved and carried out in accordance with the relevant guidelines and regulations laid down by the Institutional Ethics Committee of Postgraduate Institute of Medical Education and Research, Chandigarh, India (vide No. NK/3938/PhD). Written informed consent and assent, wherever applicable, were obtained from parents or the guardians of children prior to enrolment.

### Biochemical investigations

Plasma homocysteine (PHcy), urine homocysteine (UHcy) levels, plasma vitamin B_12,_ and plasma folate levels were measured by Advia Centaur Xp Immunoassay system using dedicated kits (Siemens, Germany). Severity of PHcy elevation was assessed using criteria described by Kang et al.^17^. Methionine and methionine/phenylalanine ratio was calculated in the dried blood spots by ABSciex 4500 triple quadrupole tandem mass spectrometer (LC–MS/MS) using Chromsystems kits, respectively (Chromsystems, Germany). Methylmalonic acid (MMA) levels in the urine were measured to rule out cases with combined homocystinuria and methylmalonic aciduria by gas chromatograph-mass spectrometer (GC–MS) using the stable isotope-labeled dilution method described by Straczek et al.^18^ with slight modifications.

### Mutation analysis

Genomic DNA of cases with suspected classical homocystinuria (n = 16) was isolated from blood collected in tubes containing ethylenediaminetetraacetic acid (EDTA) using QIAmp DNA blood mini kit (Qiagen, Germany) as per manufacturer’s instructions. PCR amplification was carried out using the primers corresponding to the coding exons and the flanking intronic regions of the *CBS* gene^19^. The PCR products were then purified using a PCR Cleanup/gel extraction kit (Nucleo-pore, India) and subjected to targeted Sanger sequencing of the *CBS* gene using Big dye terminator cycle sequencing kit on ABI Genetic analyzer 3730. *MTHFR* C677T polymorphism, a common variant associated with hyperhomocysteinemia^20^, was also analyzed through restriction fragment length polymorphism using the TaqI restriction enzyme.

## In-silico analysis

The sequences were compared with the *CBS* gene reference sequence (Genbank NM_000071.2) using the NCBI BLAST tool. Various databases, including PUBMED, 1000 genome, Human Gene Mutation Database, Exome Aggregation Consortium (ExAC), were crosschecked to determine novel variants. The damaging impact of novel missense variants was predicted through Polyphen2, PROVEAN, SIFT, Mutation Taster2, HOPE, and that of novel splice site variant through Mutation Taster 2, MaxEnt, Human Splicing Finder (HSF), Spliceman, and CRYP-SKIP software. Protein structure for c.362G>C (R121P), novel missense *CBS* variant was predicted through HOPE (https://www.cmbi.umcn.nl/hope). The protein sequence alignment of human CBS polypeptide of different species from prokaryotes to humans was performed with Clustal Omega.

### Informed consent

Informed consent (assent wherever applicable) was obtained from the parents of children studied.

## Results

Out of a total number of 16 cases subjected to targeted Sanger sequencing of the *CBS* gene, genetic testing confirmed the diagnosis in 14 cases.

## Clinical presentation (Table 1)

**Table 1:** Detail of clinical manifestations of 14 classical homocystinuria cases.

Case	1	2	3	4	5	6	7^†^	8^†^	9	10	11	12	13	14
Gender	F	F	M	M	M	F	F	M	M	F	M	M	M	F
Age at onset	3 y	9 m	1 y	5 y	8 y	2.5 y	2 y	^‡^	3 y	2.5 y	10 y	6 y	7 y	5 y
First symptom noticed	DD	Stroke	DD	Vision issues	Thrombosis	Seizures	Vision issues	^‡^	Vision issues	Stroke	Vision issues	Vision issues	Vision issues	Vision issues
Age at diagnosis (years)	11	5	7	5	8	8	5	0.5	7	2.5	10	9	8	5
Myopia	+	+	+	+	+	+	+		+	+	+	+	+	+
Ectopia lentis	+	+	+	+		+	+		+	+	+	+	+	+
Other ocular features	+ (CO)		+		+ (IR)		+ (IR,BP)		+ (Ct)			+ (Ct)		
DD	+	+	+		+		+		+		+	+		
ID	+	+^§^	+		+^§^	+			+^§^	+^§^	+^§^	+	+^§^	
Seizures		+				+						+		
Neuropsychiatric and behavioral changes	+	+	+			+			+	+	+	+		
Extrapyramidal signs	+	+										+		
Osteoporosis	+	+			+	+			+			+		
Bone deformity	+	+^¶^	+^¶^		+^¶^				+^¶^	+^¶^		+	+	
Marfanoid habitus	+	+	+		+	+			+			+		
Stroke		+								+		+		
Thrombosis		+			+				+			+		
Skin & hair changes		+			+	+	+		+	+	+		+	
PHcy levels (µmol/L)	135	224.5	160	276.43	94.9	100	54	145	121	256	284	125	163	15.6
UHcy levels (mmol/mol Cr)	41.8	18	41.8	50.3	18.6	71.4	48.79	76	18.46	31	84	76.9	62.5	4.3
DBS Met levels (ng/ml)	133	228.9	21.69	33.7	621.5	12.92	11.9	19.4	326	575	16.4	29.5	194	10.37
DBS Met/Phe ratio	2.98	6.75	0.53	0.75	10.81	0.3	0.33	0.49	9.16	10.07	0.59	1.02	8.08	0.17

*F* Female, *M* Male, *y* years, *m* months, *DD* Developmental delay, *CO* Corneal opacity, *IR* Iridodonesis, *BP* Blepharitis, *Ct* cataract, *ID* Intellectual disability, *PHcy* Plasma homocysteine, *UHcy* Urine homocysteine, *Cr* creatinine, *DBS* dried blood spots, *Met* Methionine, *Phe* phenylalanine.

^†^Siblings.

^‡^Asymptomatic.

^§^Severe intellectual disability.

^¶^Scoliosis/kyphosis.

The mean age at presentation in the genetically confirmed cases was seven years (range 2.5 year to 11 year) with a male: female ratio of 4:3. There was a wide heterogeneity in the clinical presentation, from an asymptomatic child (screened because of positive family history) to the severe forms with multisystem involvement. The two most common presenting complaints were difficulty in vision and developmental delay. The mean age at diagnosis was 6.5 year, and the majority of the cases were diagnosed after five years of age. Only one patient was diagnosed early at six months of age as a part of family screening since the elder sibling was diagnosed with homocystinuria. Myopia was observed in 13/14 cases (93%), and ectopia lentis was observed in 12/14 cases (86%). Decreased near vision was the primary complaint in 69% of the cases with myopia. Other rare ocular symptoms were iridodonesis (3/14), phacodonesis (1/14), cataract (1/14), and blepharitis (1/14). Ten children (76%) had an intellectual impairment. Osteoporosis was observed in 6/14 (43%) cases. Tall stature was observed in 5/14 (36%) cases; however, short stature was also observed in 3/14 (21%) cases. Stroke and thrombosis were observed in 5/14 (36%) cases.

### Biochemical investigations (Table 1)

The mean baseline PHcy level among cases was 153.8 µmol/L (range 15.6–284). Eleven out of 14 (78.5%) cases had severely elevated PHcy (> 100 µmol/L) levels. The baseline values of urine homocysteine and dried blood spot methionine levels were not available for the majority of the cases, as they had already been initiated on treatment based on the elevated plasma homocysteine levels during their initial visits. The mean UHcy level was 43.9 mmol/molCr (range 4.3–84) and the mean methionine level was 159.96 µmol/L (range 10.37–621.5). Six cases (47%) had high (> 40 µmol/L), and 8/14 (53%) cases had low-normal to normal (< 40 µmol/L) methionine levels. The mean methionine/phenylalanine ratio was 3.72 (range 0.17 to 10.81) and 50% cases had a high ratio (cut-off = 0.99).

### Genetic analysis (Table 2)

**Table 2:** Details of *CBS* gene variants and *MTHFR* C677T polymorphism of 14 classical homocystinuria cases.

Case	*CBS* exon	cDNA nucleotide change	Protein change	Type	Reported	*MTHFR* C677T status
1	10	c.1136G>A	p.R379Q	MISSENSE	Reported^21^	C/C
	6	**c.736 + 2T>C**	**?**	**SPLICING**	**Novel**	
2	13	**c.1397C>A**	p.S466*	**NONSENSE**	**Novel**	C/C
3	7	c.785C>T	T262M	MISSENSE	Reported^22^	C/T
4	3	c.430G>A	p.E144K	MISSENSE	Reported^23^	C/C
	1	c.19del	p.Q7Rfs*75	FRAMESHIFT-DEL		
5	7	**c.825C>A**	p.C275*	**NONSENSE**	**Novel**	C/T
	1	c.19del	p.Q7Rfs*75	FRAMESHIFT-DEL	Reported^24^	
6	4	c.518_520del	p.M173del	INFRAME-DEL	Reported^25^	C/C
7^†^	9	c.992C>T	p.A331V	MISSENSE	Reported^23^	C/T
8^†^	9	c.992C>T	p.A331V	MISSENSE	Reported^23^	C/T
9	9	c.982G>A	p.D328N	MISSENSE	Reported^26^	C/C
10	5	**c.566_567del**	p.V189Efs*49	**FRAMESHIFT-DEL**	**Novel**	C/C
	3	**c.362G>C**	**p.R121P**	**MISSENSE**	**Novel**	
11	3	c.430G>A	p.E144K	MISSENSE	Reported^23^	C/C
	1	c.19del	p.Q7Rfs*75	FRAMESHIFT-DEL	Reported^24^	
12	4	c.518_520del	p.M173del	INFRAME-DEL	Reported^25^	C/T
	3	**c.402del**	p.T135Rfs*2	**FRAMESHIFT-DEL**	**Novel**	
13	11	**c.1217del**	p.K406Sfs*18	**FRAMESHIFT-DEL**	**Novel**	C/T
14	15	c.1642C>T	p.R548W	MISSENSE	Reported^27^	C/C

*C/C* MTHFR 677CC wild-type genotype, *C/T* MTHFR 677CT heterozygous genotype.

^†^Siblings.

**Figure 1:**
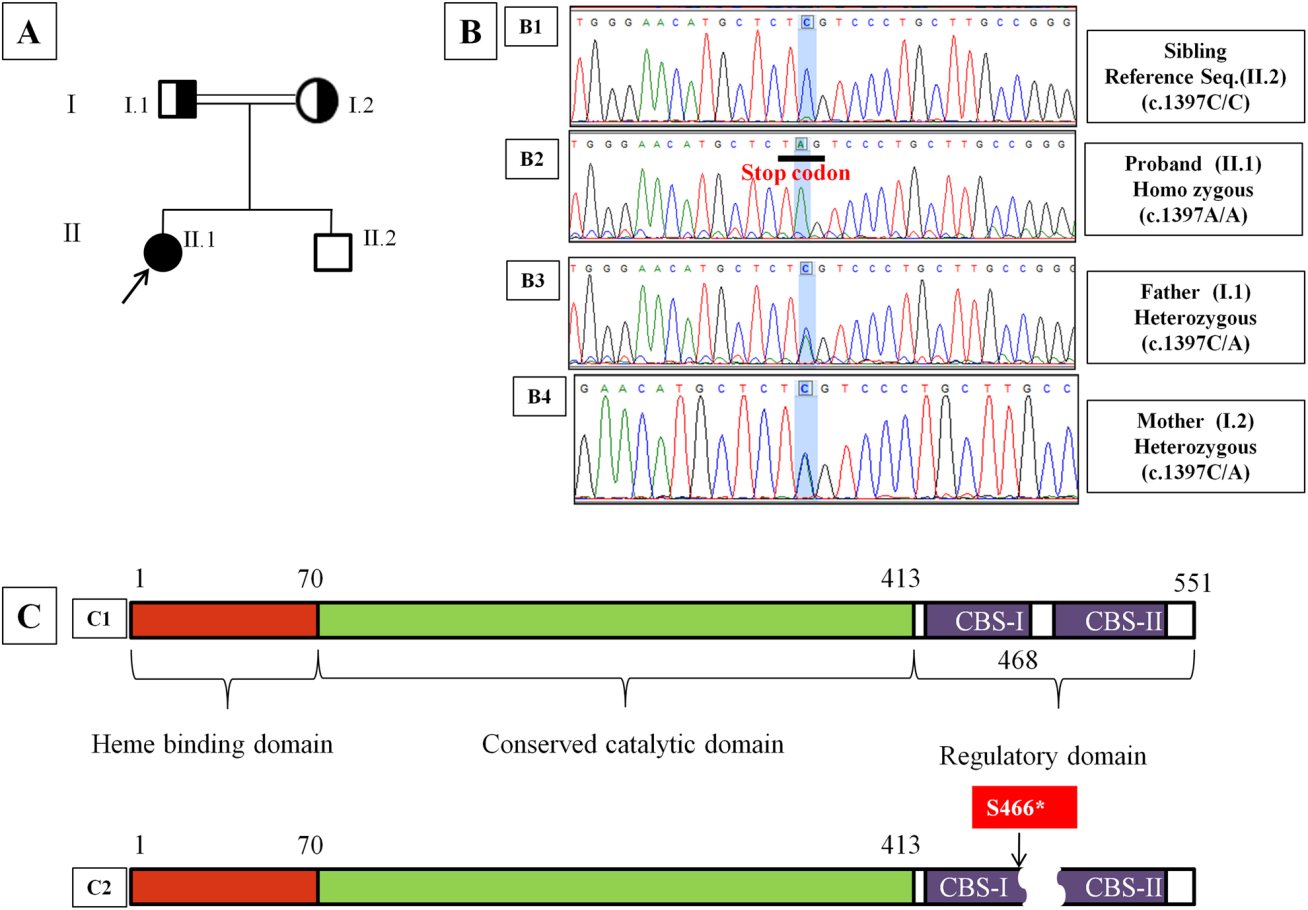
(**A**) Pedigree of case 2 (P-2). (**B**) Sequencing chromatograms of (**B1**) of unaffected sibling showing peak for wild type nucleotide cytosine (**C**) at 1397 position of CBS cDNA representing wild type genotype as the reference sequence, (**B2**) Proband (case 2) showing single peak for adenine (**A**) at 1397 representing homozygous substitution of C by A (c.1397A/A), (**B3**, **B4**) showing double peaks (both **A** and **C**) in DNA of father and mother representing heterozygous genotype (i.e. c.1397C/A), respectively. (**C**) Schematic diagram showing (**C1**) wild type CBS polypeptide, (**C2**) Truncated CBS polypeptide due to nonsense variant (c.1397C>A) resulting in substitution of serine (S) at 466 amino acid position by stop codon, TAG (*).

A total of 15 different variations were identified in 14 cases from 13 unrelated Indian families with homocystinuria. Of these, four children belonged to consanguineously married parents and were homozygotes for *CBS* variants. Of the 15 variants, seven were novel. Three out of the seven novel variants were frame-shift deletions (c.566_567del, c.402del, c.1217del), two were nonsense variants (c.825C>A, c.1397C>A) (Fig. 1), one was missense (c.362G>C) and one was a splicing variant (c.736 + 2T>C). Seven of the cases (i.e. cases 2,3,6,7,8,9, and 13) had homozygous *CBS* variants. Another six cases (cases 1, 4, 5, 10, 11, and 12) had compound heterozygous variants. In one of the cases (case 14), we could not find any *CBS* variant in the second allele (heterozygous). The eight variants c.1136G>A c.785C>T, c.430G>A c.19del, c.518_520del, c.992C>T, c.982G>A and c.1642C>T had already been reported (Table 2) from other population cohorts. The *CBS* gene variants were distributed throughout the gene with maximum variants in the exon 3 (Supplementary Fig. 1). We did not find any pathogenic variants in the exons 2, 8, 12, and 14. Along with the *CBS* gene variants, *MTHFR* polymorphism C677T in the heterozygous state (677C/T) was observed in six out of the 14 cases.

### ***In-silico*** analysis of the ***CBS*** gene variants (Table 3)

**Table 3:** In silico analysis: scores and prediction of novel missense *CBS* variant c.362G>C (R121P) and splice site variant (c.736 + 2T>C).

Variant	In silico tool	Score	Prediction
c.362G>C (p.R121P)	PROVEAN	− 6.96 (cut off = − 2.5)	Deleterious
	SIFT	0.000 (cut off = 0.05)	Damaging
	Polyphen2	1.000 (cut off = 0.50)	Possibly damaging
	Mutation taster	103	Disease causing
	CADD	27.1	Damaging
c.736 + 2T>C	MaxEnt	93.83	Altered or broken wild type donor splice site
	Human Splicing Finder	31.82	Broken wild type donor splice site
	Splice man	63*	Altered or broken wild type donor splice site
	Mutation Taster	NA	Disease-causing: likely to disturb normal splicing
	CRYP-SKIP	0.23^#^	Exon skipping

All the seven novel variants were predicted to be deleterious using Mutation Taster 2.0. The three frameshift deletions and two nonsense variants found in the *CBS* gene were predicted to cause premature termination. This resulted in either a nonsense-mediated decay of the CBS polypeptide^28,29^ or a truncated CBS polypeptide depending upon the location of variants^30^. As per the recommendations of the American College of Medical Genetics, such variants are considered to be potentially pathogenic^31^. Wild type donor splicing site was predicted to be altered/broken in the novel variant, c.736 + 2T>C described as a donor splice site variant through various softwares (details in Table 3).

**Figure 2:**
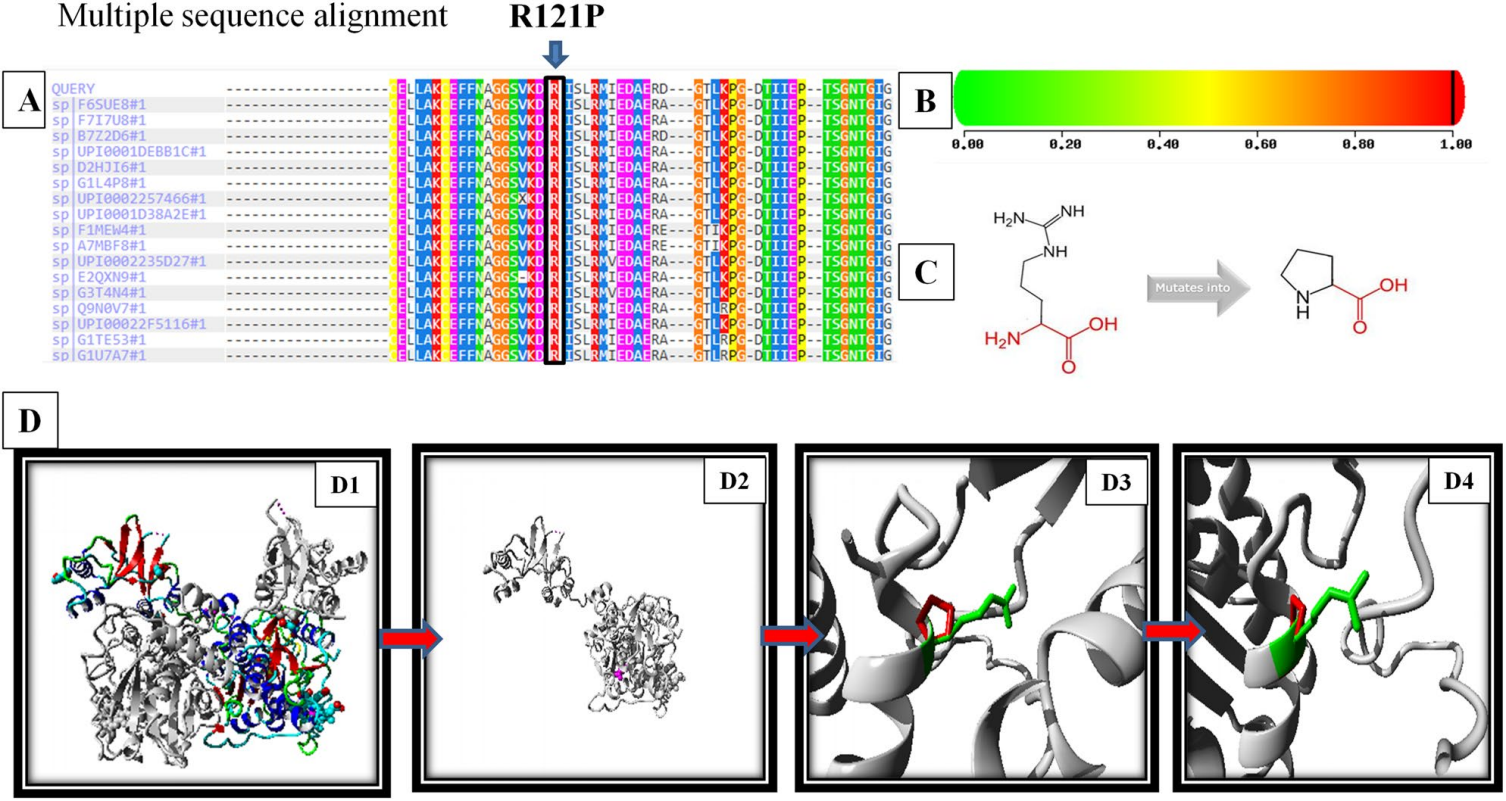
Prediction of damaging effect of novel *CBS* missense variant (c.362G>C). (**A**) Multiple sequence alignment of the amino acid sequence near mutant location generated through PolyPhen-2. (**B**) Score of PolyPhen-2 at red zone (i.e. 1.00) indicating probably damaging effect of c.362G>C (R121P) variant. (**C**) Structure of wild type residue (Arginine) and mutated residue (Proline) showing the size, structural and side chain differences of the two residues (**D**) Protein structure prediction using HOPE software (**D1**) wild-type CBS protein, (**D2**) mutated protein with novel misense R121P variant, (**D3**, **D4**) close view of variation (wild type amino acid, arginine in green and mutated residue, proline in red).

On the evaluation of the conservation status of c.362G > C (R121P) novel missense variant, only the wild type arginine (R) residue was found at 121 positions across all species. This suggested that the novel missense variant (R121P) occurred at a highly evolutionarily conserved residue and functionally active residual domain in the protein (Fig. 2). Protein structure for R121P predicted through HOPE revealed that mutant residue (proline) was smaller in size and neutral in charge, whereas the wild-type (arginine) residue has a larger side chain and is positively charged. In addition, the mutant residue was more hydrophobic than the wild-type residue. The wild-type residue forms a hydrogen bond with glutamic acid at position 110 and 239, and threonine at position 235. The size difference between the wild-type and the mutant residue did not allow the new residue to be in the correct position to make the same hydrogen bond as the original wild-type residue did. The difference in the hydrophobicity would have affected the hydrogen bond formation. The wild-type residue forms a salt bridge with aspartic acid at position 86 and glutamic acid at position 110 and 239. The difference in charge will disturb the ionic interaction made by the original, wild-type residue. Based on the above information, this variant was assumed to be damaging to the protein.

## Discussion

In the present study, we report the clinical and biochemical profiles and the spectrum of *CBS* gene variations in North Indian children presenting with classical homocystinuria. The disorder typically presents with elevated methionine levels; however, only 47% of our cases showed high methionine levels (> 40 µmol/L). Baseline methionine levels were available only in 29% of the cases, which were newly diagnosed. Of the eight cases with a normal methionine level, four had *MTHFR* C677T polymorphism in the heterozygous state (677C/T genotype). The possible reasons for lower methionine levels in our cases could be because the children were already on treatment, had a coexisting vitamin B_12_ deficiency, or associated with *MTHFR* 677C/T genotype^9^.

In our study, one case had a single heterozygous *CBS* variant, while the remaining 13 cases were either homozygous or compound heterozygous for *CBS* variants. The majority (85%) of these homozygous/compound heterozygous patients had a severe elevation, while the rest had a moderate elevation of PHcy. The patient with the single heterozygous variant had a mild elevation of PHcy with a milder clinical presentation. In contrast to other cases where multisystem involvement had been observed, this case had presented only with myopia and lens dislocation. We did not detect a *CBS* variant in the coding region of the second allele in this case. However, the promoter region, deep intronic variants, and untranslated regions of the *CBS* gene have not been screened. Even though the CBS defect follows an autosomal recessive inheritance, the milder phenotypic expression in this case maybe attributed to heterozygosity. A few studies have already documented the effect of the heterozygous genotype of the *CBS* gene in the manifestation of the disease, although a milder one^9,32,33^. In other words, even children with mild hyperhomocysteinemia should be evaluated with genetic workup after cobalamin deficiency has been ruled out in them.

The majority of the variants identified were missense (46%), followed by frameshift deletions (27%). Though I278T and G307S variants found in the exon 8 were previously reported to be the most common variants from different populations including Europe, the US, Australia^9,23^, and Ireland (unusual high frequency of G307S in 71% Irish patients), neither of these two variants were found in our study group or in the cohort reported from China^10^. We did not find even the third most frequently known variant (i.e., IVS11-2A > C) in intron 11^23^ in any of our homocystinuria children.

Children with classical homocystinuria cases can be misdiagnosed as harboring cobalamin defects. Case 10 had a typical and severe phenotype with an acute ischemic stroke at 2.5 years of age, followed by myopia, lens dislocation, pectus carinatum, kyphoscoliosis, short stature, intellectual disability, fair skin, malar flush, hypopigmented brittle, thin hair, and severely elevated levels of homocysteine (122 µmol/l) despite the treatment. This child was initially misdiagnosed as having combined homocystinuria and methylmalonic aciduria due to the massive excretion of MMA in the urine. The methionine levels were not available at that time. The diagnosis of classical homocystinuria was made during the subsequent follow-up visits when elevated homocysteine and methionine levels were noted with normalization of MMA levels (on treatment with vitamin B_12_). Therefore, in countries like India, where vitamin B_12_ deficiency is quite common, it becomes imperative to correct the vitamin B_12_ deficiency before assigning the diagnosis of isolated homocystinuria. This child was found to be compound heterozygous for two novel variants, i.e. (V189Efs*49; c.566_567del) and (R121P; c.362G>C). As per the structure and function prediction by HOPE, the variant could have disrupted the interaction among the residues and, subsequently, the protein function. Two other variants at the same position, i.e., c.362G>A (R121H) and c.362G>T (R121L), had previously been reported to be mild in the African/American and of unknown severity in the Chinese population, respectively^23^.

The c.736 + 2T>C (IVS7 + 2T>C), a novel splicing variant, was observed along with R379Q (c.1136G>A) in case 1. Approximately 99% of the introns contain canonical dinucleotides GT at positions + 1 and + 2 of 5′ splice site and most of the pathogenic splice site variations at donor site lead to noncanonical dinucleotides instead of GT. However, there are reports of non-canonical splice site usage in ~ 1% of all human 5′ splice sites. So, in a fraction of cases, a T>C change at + 2 position of 5′ splice site could still result in normal splicing ^34^. The child with c.736 + 2T>C had a severe phenotype with multisystem involvement and a remarkable sibling history (elder sibling’s death with similar complaints). The phenotype of our case, however, suggests that the variant c.763 + 2T>C would result in missplicing. This would require assessing the splice variants by expression minigenes, as previously reported^35,36^. R379Q was previously reported in a Spanish patient, and the variant was predicted to affect the dimer formation or the stability of CBS protein. Urreizti et al. found this R379Q variant in a compound heterozygous form, and the disease phenotype was also reported to be severe, but an exact comparison cannot be made among the two cases because of compound heterozygosity in the patients^21^.

Case 5 presented with severe and typical classical homocystinuria and harbored compound heterozygous [C275* (c.825C>A), Q7Rfs*75 (c.19del)] variants. The c.19del variant was previously reported in the ExAC browser in two South Asian alleles in heterozygous form, but its association with homocystinuria has never been reported earlier. Instead, Kozich et al. reported c.19ins, resulting in frameshifting in a case with a severe phenotype^37^. Similar to patient 10, it was also misdiagnosed initially due to an elevated MMA found in the urine. Both the variants, in this case, were predicted to result in premature termination of the CBS polypeptide with 274 amino acids (c.825C>A) and 80 amino acids (c.19del) in comparison to the 551 amino acids in the wild type CBS polypeptide. Similarly, case 12 presented with two deletion variants (c.402del), out of which M173del (c.518_520del) inframe deletion has been previously reported^25^. Though the majority of their study population^25^ was Spanish and Argentinian, they found a homozygous M173del variant in an Indian patient, suggesting its association with the Indian origin. The variant was characterized in the *E. coli* expression system and was found to have null CBS enzyme activity, whereas the disease phenotype was reported to be mild. In contrast, the disease phenotype of our case was severe, which may be attributed to the pathogenic frameshift deletion (T135Rfs*2) in his second allele. At presentation, both cases 5 and 12 had severe clinical (ectopia lentis, mental retardation, and deep vein thrombosis) and biochemical phenotypes, supporting the pathogenicity of these deletion variants.

**Figure 3:**
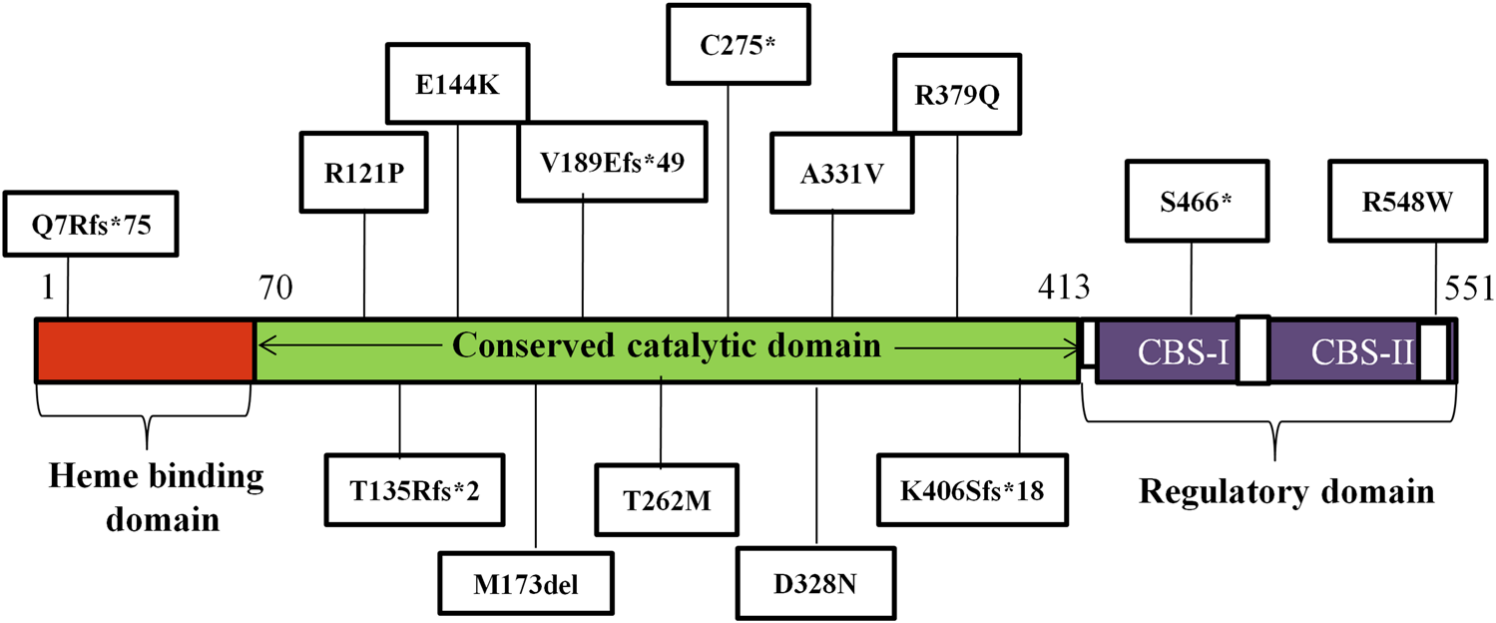
Schematic diagram representing distribution of *CBS* gene variants among three domains of CBS polypeptide.

The c.982G>A (D328K) variant was found to have clinical as well as severe biochemical phenotype (case 9), similar to the findings of Silao et al. in a 16-year-old Filipino patient^26^. Interestingly, both cases also had cataracts, which is not a very common finding in homocystinuria. In our case, the child had severe mental retardation (IQ = 28, SQ = 22) along with aggressive behavior in comparison to the mild to moderate IQ in the Filipino case. Among the vascular complications, our case had a history of deep vein thrombosis, whereas peripheral arterial occlusive disease was reported in the Filipino case. The c.992C>T variant was found in two siblings (cases 7 and 8), which was earlier reported by Kruger and Cox^38^. The younger sibling is seven years old and is asymptomatic. He was screened due to the family history, and the treatment was started at the age of one year. This case highlights the importance of early diagnosis and treatment either by newborn screening or family screening for homocystinuria. Most of the variants (12/15) were observed in the most conserved region, i.e., the catalytic domain of the CBS enzyme and three variants in each of the heme-binding site, CBS-I regulatory domain, and C-terminal region (Fig. 3). The remaining two cases screened negative for *CBS* variants and underwent targeted next-generation sequencing for a gene panel with nine genes associated with homocystinuria (*CBS, MTR, MTRR, MTHFR, MMACHC, MMADHC, ABCD4, LMBRD1,* and *HCFC1)*. One of these cases was found to have a novel heterozygous splice site variant in the *ABCD4* (data not shown) and a heterozygous C677T variant in the *MTHFR* gene. In the second case, we could not detect any variant in any of the nine genes mentioned above. The case presented typically with Marfanoid features and could be a case of Marfan syndrome with homocystinuria; however, we did not study the gene for Marfan syndrome.

It is difficult to predict the phenotype from the genotype in the cases due to high genotypic heterogeneity and compound heterozygosity. Except for one case, all the combinations of variants in the cases are unique. Hence, any general statement could not be drawn from these individual cases. The c.1397C>A, c.[825C>A, 19del], c.982G>A, c.[566_567del, 362G>C], and c.[518_520del, 402del] genotypes have been observed with life-threatening events such as thromboembolism and stroke. As all the variants were highly suggestive of being pathogenic, further functional studies might help in investigating the role of the novel variants at the protein level to understand the underlying mechanism of disease and novel targets for the possible therapeutic role. In lieu of a wide spectrum of *CBS* gene variants from our study, population-specific studies may be required to further identify additional *CBS* gene variants prevailing in different regions of India. Institution of the newborn screening program is of utmost importance for an early diagnosis since delayed diagnosis results in significant morbidity and compromised quality of life.

## Conclusion

Our study identified the spectrum of variants prevailing in the *CBS* gene responsible for classical homocystinuria from India. To the best of our knowledge, this is the first study to provide a genetic characterization of homocystinuria in India. Due to the wide genotypic heterogeneity found in our cases and the compound heterozygosity in nearly half of the cases, we could not establish genotype–phenotype correlations from individual patients. Myopia and ectopia lentis were observed as the most consistent clinical findings. Biochemical and genetic analysis has allowed us to diagnose classical homocystinuria accurately correctly. This study also reports the first case of the rare *ABCD4* defect of cobalamin metabolism from India. Future studies should focus on new therapeutic targets based on the type of genetic variations.

## Supplementary information

Supplementary Information.

## Data Availability

The data that support the findings of this study are available from the corresponding author upon reasonable request.
