# Supplementary material for: Seven novel genetic variants in a North Indian cohort with classical homocystinuria
Source: Sci Rep. 2020 Oct 14;10:17299. doi: 10.1038/s41598-020-73475-5 (PMC7560719; doi:10.1038/s41598-020-73475-5)
Supplement: Supplementary file 1 — Supplementary Information. [file 41598_2020_73475_MOESM1_ESM.pdf]

Seven novel genetic variants in a North Indian Cohort with classical Homocystinuria

Rajdeep Kaur[1], Savita V Attri[1*], Arushi G Saini[1], Naveen Sankhyan[1], Satwinder Singh[1], Mohammed Faruq[2], VL Ramprasad[3], Sheetal Sharda[3], Sakthivel Murugan[3]

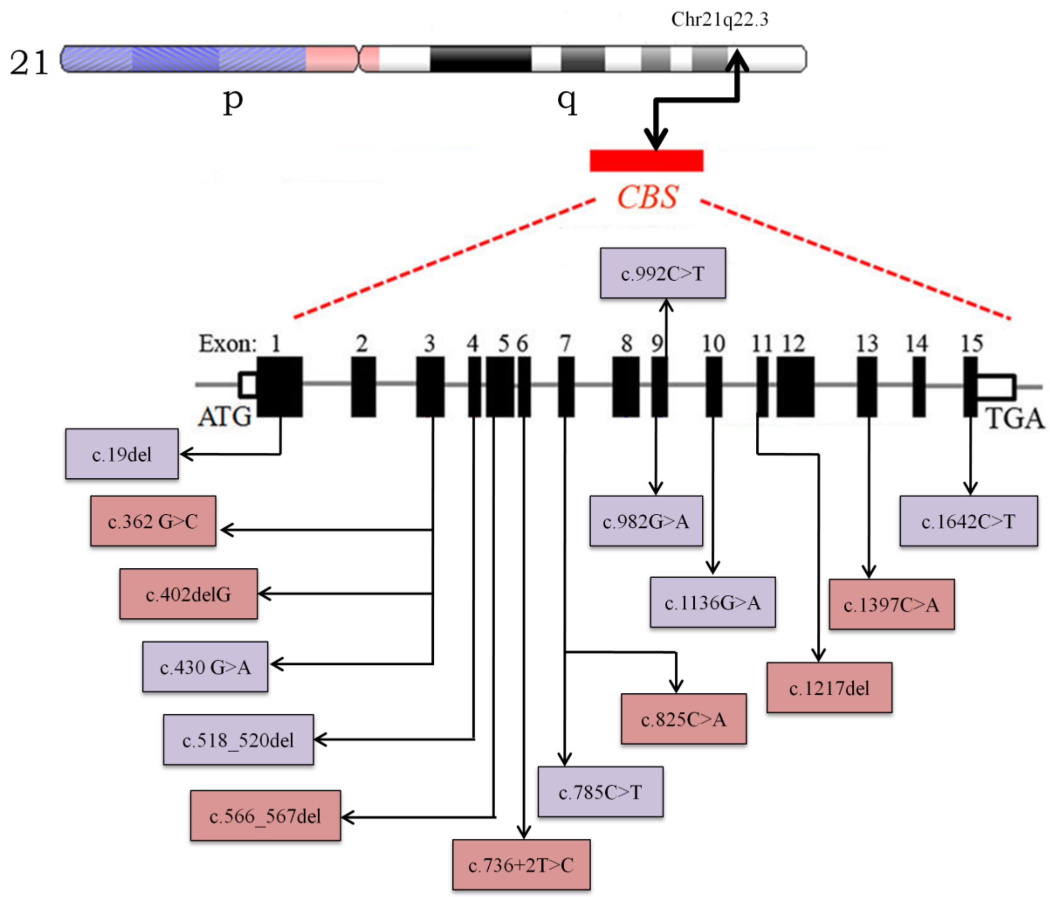

Supplementary Figure 1: Schematic diagram representing exon-wise distribution of *CBS* gene variants chromosomal (Red boxes – Novel variants, purple boxes - reported/ known variants).
